# The Amyloid Precursor Protein (APP) Does Not Have a Ferroxidase Site in Its E2 Domain

**DOI:** 10.1371/journal.pone.0072177

**Published:** 2013-08-19

**Authors:** Kourosh Honarmand Ebrahimi, Christian Dienemann, Sandra Hoefgen, Manuel E. Than, Peter-Leon Hagedoorn, Wilfred R. Hagen

**Affiliations:** 1 Department of Biotechnology, Delft University of Technology, Delft, The Netherlands; 2 Leibniz Institute for Age Research-Fritz Lipmann Institute (FLI), Protein Crystallography Group, Jena, Germany; Torrey Pines Institute for Molecular Studies, United States of America

## Abstract

The ubiquitous 24-meric iron-storage protein ferritin and multicopper oxidases such as ceruloplasmin or hephaestin catalyze oxidation of Fe(II) to Fe(III), using molecular oxygen as oxidant. The ferroxidase activity of these proteins is essential for cellular iron homeostasis. It has been reported that the amyloid precursor protein (APP) also has ferroxidase activity. The activity is assigned to a ferroxidase site in the E2 domain of APP. A synthetic 22-residue peptide that carries the putative ferroxidase site of E2 domain (FD1 peptide) has been claimed to encompass the same activity. We previously tested the ferroxidase activity of the synthetic FD1 peptide but we did not observe any activity above the background oxidation of Fe(II) by molecular oxygen. Here we used isothermal titration calorimetry to study Zn(II) and Fe(II) binding to the natural E2 domain of APP, and we employed the transferrin assay and oxygen consumption measurements to test the ferroxidase activity of the E2 domain. We found that this domain neither in the presence nor in the absence of the E1 domain binds Fe(II) and it is not able to catalyze the oxidation of Fe(II). Binding of Cu(II) to the E2 domain did not induce ferroxidase activity contrary to the presence of redox active Cu(II) centers in ceruloplasmin or hephaestin. Thus, we conclude that E2 or E1 domains of APP do not have ferroxidase activity and that the potential involvement of APP as a ferroxidase in the pathology of Alzheimer’s disease must be re-evaluated.

## Introduction

**Figure 1:**
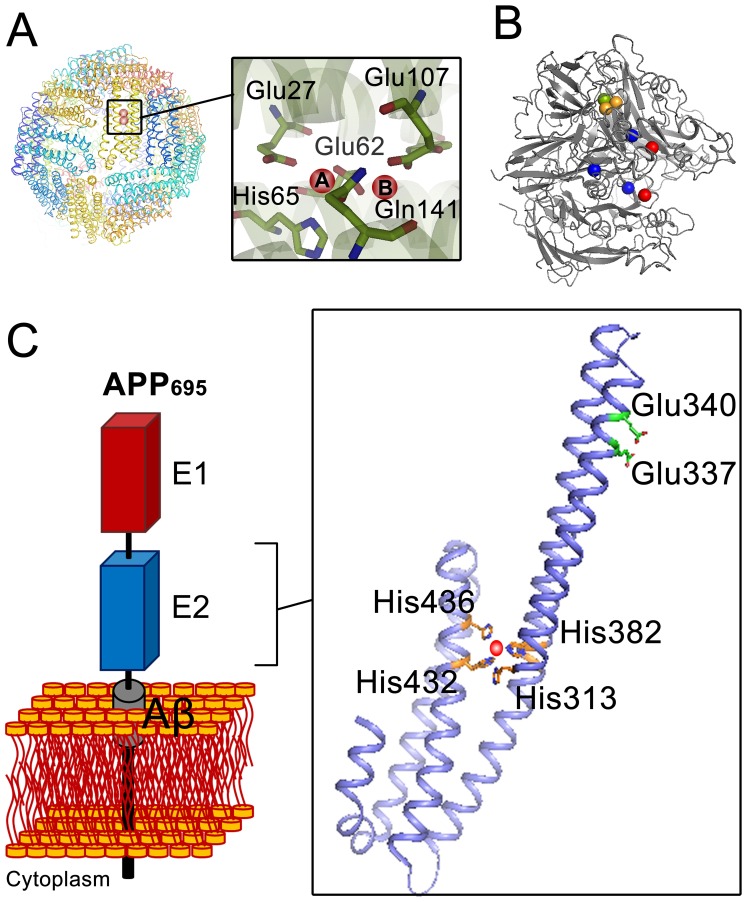
Comparison of the X-ray crystal structure of proteins with ferroxidase activity with that of the E2 domain of amyloid precursor protein (APP). (A) Quaternary structure of 24-meric ferritin (HuHF, PDB code 2FHA) showing the position of the diiron binding site where the ferroxidase reaction occurs. The two iron binding sites are marked with A and B. (B) Structure of the multicopper oxidase ceruloplasmin (PDB code 1KCW). Ceruloplasmin contains of three type I copper centers (blue sphere), one type II copper center (green sphere), and one type III copper center (orange sphere). Type II and III centers together form a trinuclear copper center which is responsible for four electron oxidation of molecular oxygen to water. Red spheres in the structure show other possible metal binding sites. (C) A schematic representation of the APP and X-ray structure of the E2 domain of APP_695_ (PDB 3UMH). The structure shows the specific Cu(II) (red sphere) binding site (M1 site) with four histidines (His313, His382, His432, and His436) as coordinating residues. Glu337 and Glu340 are the putative ligands of the previously defined ferroxidase site in the E2 domain of APP [21]. The numbering of the residues is based on APP_695_.

Oxidation of Fe(II) to Fe(III) is an essential reaction in cellular iron homeostasis. Under physiological conditions this reaction can occur spontaneously in the presence of oxidants such as molecular oxygen or hydrogen peroxide. Spontaneous (i.e. not biologically catalyzed) oxidation of Fe(II) produces Fe(III) and reactive oxygen species (ROS). Fe(III) is essentially insoluble under physiological conditions, with a solubility of 10^−10^ M [1], and will therefore precipitate, whereas ROS such as the hydroxyl radical will react uncontrollably with many components of the cell. To prevent formation of these toxic products and to keep the iron in a soluble form for cellular usage, proteins evolved to carry out controlled catalytic oxidation of Fe(II) to Fe(III) in the ferroxidase reaction. The proteins for which ferroxidase activity has been established can be divided into two main groups: (i) members of the ferritin superfamily [2], [3] including ferritin (Figure 1A), bacterioferritin, and Dps (DNA binding protein from starved cells), and (ii) multicopper oxidases [4] such as ceruloplasmin [5], [6] (Figure 1B) or hephaestin [7], [8]. The ferroxidase activity of proteins in the ferritin superfamily is essential for controlling the intracellular concentration of Fe(II) or for protection of DNA from reactive oxygen species. For example ferritin and bacterioferritin oxidize excess Fe(II) and store the resulting Fe(III) product in a non-toxic form [9], [10]. The ferroxidase activity of multicopper oxidases such as ceruloplasmin appears to be essential for transport of iron across cellular membranes [11]–[15].

In ferritin the ferroxidase reaction occurs in a diiron binding site, the ferroxidase center, with a highly conserved tyrosine in the vicinity of this site essential for the catalytic activity [16] (Figure 1A). The Fe(II) binds to this center and reacts with molecular oxygen under formation of either hydrogen peroxide or water [16]. The metastable Fe(III) product leaves the ferroxidase center and enters the protein cavity upon arrival of incoming Fe(II) ions [17], [18]. In some multicopper oxidases such as ceruloplasmin and haphaestin the ferroxidase reaction appears to occur via outer-sphere electron transfer [4]. It is proposed that electrons are transferred from the Fe(II) ions bound to the protein to a type I copper center and then to a trinuclear copper center where molecular oxygen is reduced to water (Figure 1B) [4]. Possible Fe(II) binding sites have been identified in ceruloplasmin [19], [20]. The resulting Fe(III) product in these ferroxidases is proposed to be scavenged by an Fe(III)-binding protein such as transferrin to prevent precipitation of Fe(III) products.

Recently a ferroxidase activity has also been reported for the amyloid precursor protein (APP) [21] (Figure 1C). APP is a transmembrane protein which consists of two extracellular domains known as the E1 and E2 domains, a short transmembrane section containing part of the Aβ peptide, and a small interacellular domain (AICD) [22]. Alternative splicing of exon regions of the APP mRNA creates APP- isoforms with different amino acid lengths [23], [24]. APP is of special interest because of its possible role in Alzheimer’s disease [25]. Duce et al. [21] have recently reported that the E2 domain of APP has a putative ferroxidase site, which behaves like the ferroxidase center of ferritins. It was observed that the E2 domain in the presence of the E1 domain has ferroxidase activity equal to that of the full APP-ectodomain and comparable to that of ceruloplasmin. The ferroxidase activity of the E2 domain was inhibited by Zn(II) like that of ferritin [26]–[28]. Based on these findings it has been proposed that the ferroxidase activity of APP in Alzheimer’s disease has the same function as the ferroxidase activity of ceruloplasmin coupled to iron-export activity of ferroportin [14]: the Fe(II) ion that is exiting ferroportin binds to the ferroxidase site of APP, it is oxidized by molecular oxygen, and the resulting Fe(III) product is then scavenged by the ferric binding protein, transferrin. In individuals with Alzheimer’s disease Zn(II) would bind to the ferroxidase site in the E2 domain of APP and inhibits its ferroxidase activity, resulting in accumulation of intracellular Fe(II) and subsequent oxidative damage of the cells. Duce et al. [21] used an unfitting structural assignment as discussed previously [29], and they applied the Fe(III)-transferrin colorimetric assay to measure the ferroxidase activity of APP, its E2 domain, and of the E2-domain derived synthetic 22-residue peptide FD1. Previously we tested the ferroxidase activity of the FD1 peptide by following the production of Fe(III) with the transferrin assay, and the consumption of molecular oxygen amperometrically. We found that the FD1 peptide does not have any ferroxidase activity and that Zn(II) interferes with the transferrin assay [29]. In the present study we re-evaluate the described ferroxidase activity of the E2 domain of APP and the effect of the E1 domain on this activity. We show that consistent with our previous results for the synthetic FD1 peptide [29] the E2 domain of APP does not bind Fe(II) and does not have a ferroxidase activity either in the presence or in the absence of the E1 domain.

## Results and Discussion

### The E2 Domain Binds Cu(II)

**Figure 2:**
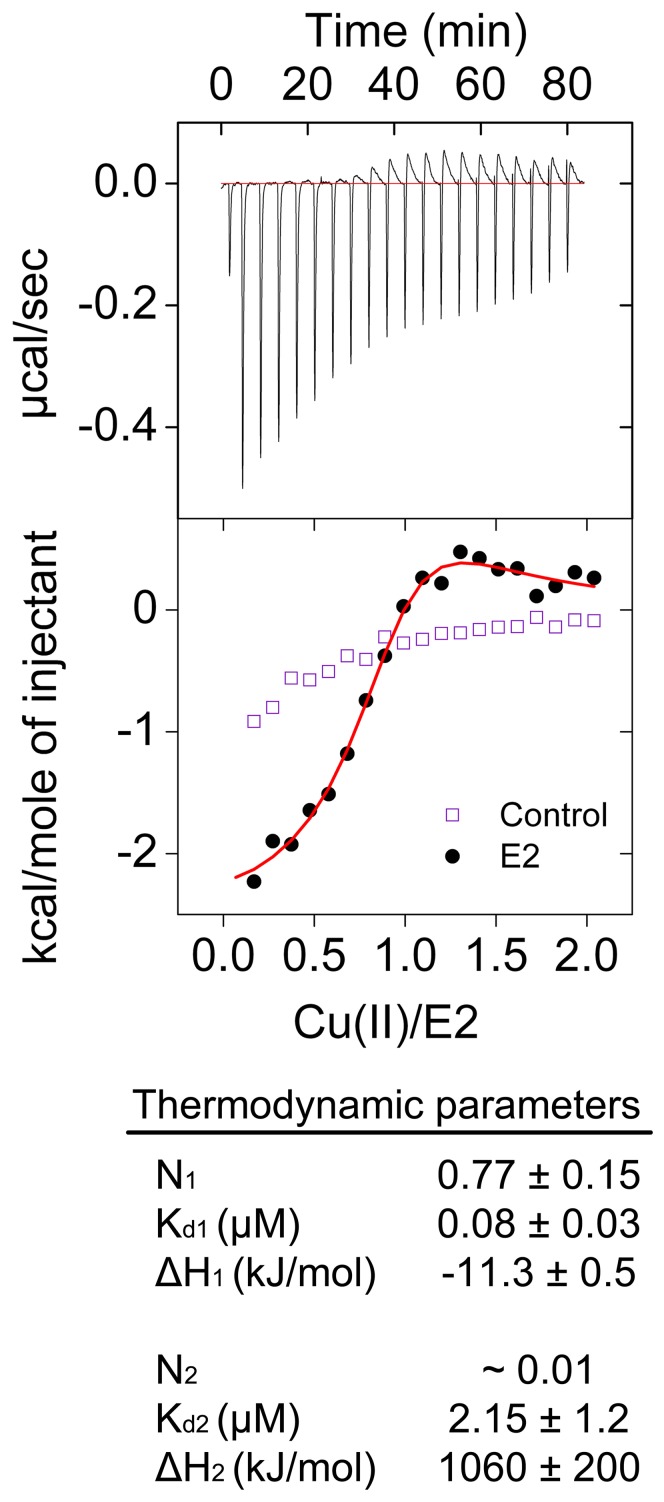
Cu(II) binding to the E2 domain of APP measured by ITC. We measured binding of Cu(II) to the E2 domain in non-coordinating buffer using isothermal titration calorimetry (ITC). Concentration of E2 domain in the cell was 26.5 µM and that of Cu(II) in the syringe was 1.27 mM. Measurements were performed at 25°C in 100 mM Mops 150 mM NaCl, pH 7.0. A model with two independent binding sites was required to obtain a fit to the data of integrated heat of binding. *The stoichiometry of the second binding event could not be determined with statistical significance. The data represent the average of two experiments ± standard deviation.

Before measuring ferroxidase activity and Fe(II) binding of the E2 domain of APP, we measured binding of Cu(II) to the E2 domain. Cu(II) binding was used to test the correct folding state of the protein because the APP and its E2 or E1 domains do not have any established catalytic activity. Protein crystallography and a number of biochemical and biophysical studies were used before to ascertain the functional fold of the used recombinant protein [28]. Its purification was also assessed by SDS-PAGE analysis (Figure S1). As four histidines must come together from sequentially distant places in primary structure to form the M1-site of the E2 (Figure 1), binding of Cu(II) to the E2 domain is probably one of the best measurements to analyze its correct three-dimensional fold. Using isothermal titration calorimetry (ITC) it has been previously reported that the E2 domain of APP binds Cu(II) with a stoichiometry of circa 0.7 Cu(II) per E2 domain and a dissociation constant of 0.013±0.005 µM [30]. Those measurements were performed in Tris buffer at pH 7.3 (Tris is a competing ligand) to eliminate any low-affinity binding event and the results were corrected for the Cu(II) binding to Tris. We measured binding of Cu(II) to the E2 domain of APP using ITC in Mops buffer pH 7.0 (Figure 2). A model with two independent binding sites was required to obtain a fit to the data of integrated heat of binding. It resulted in two binding events (Figure 2): one binding event with a stoichiometry of 0.77±0.15 Cu(II) per E2 domain and a dissociation constant of 0.08±0.03 µM, and a second low affinity binding event. The stoichiometry of the second binding event could not be determined with precision due to its low affinity; however, this binding event was required to obtain a fit to the experimental data. The thermodynamic parameters of the first binding event in Mops buffer (Figure 2) are within experimental error identical to the previously published results when corrected for Tris binding [30]. The second low affinity binding event is possibly due to non-specific binding of Cu(II) to the E2 domain of APP or Cu(II) induced intermolecular interactions between the E2 domains.

**Figure 3:**
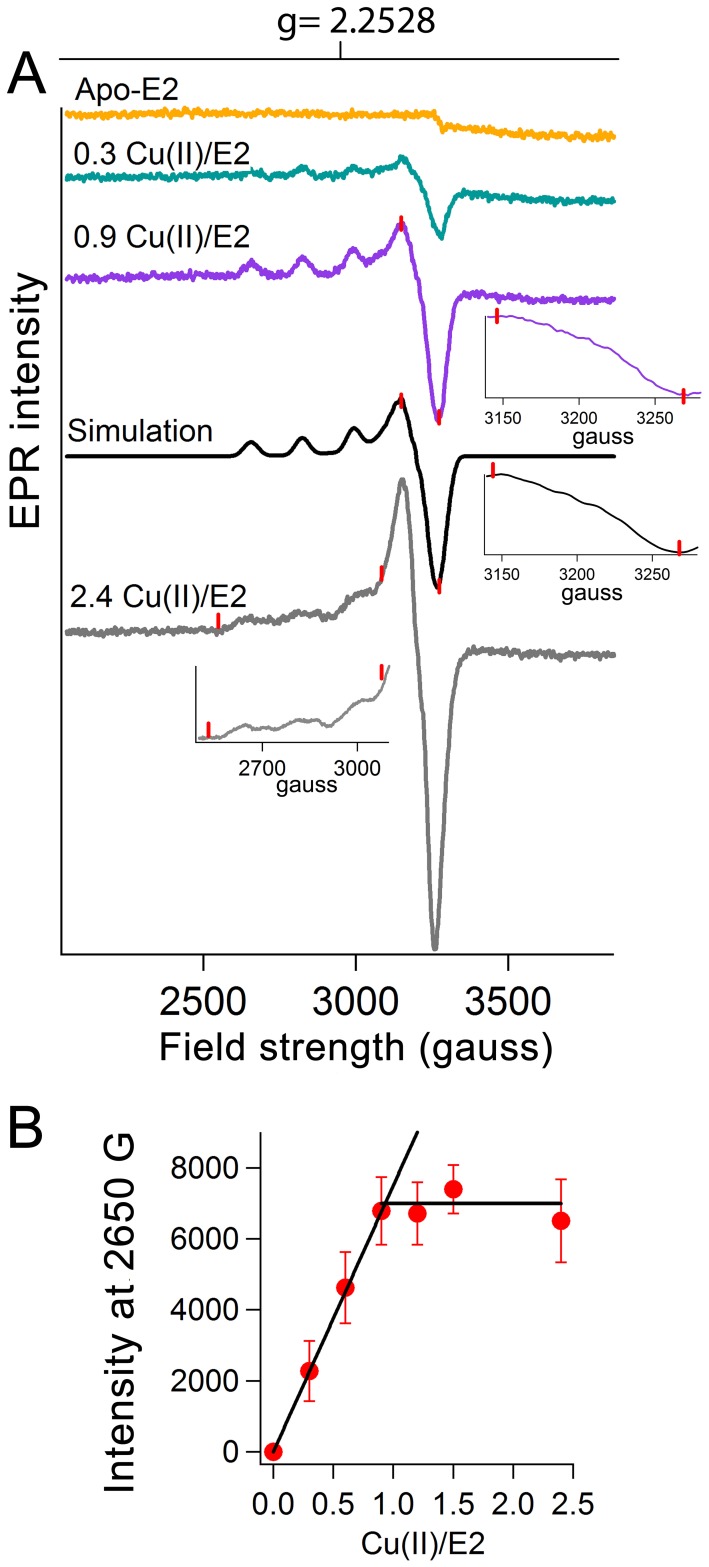
Cu(II) binding to the E2 domain of APP measured by EPR spectroscopy. (A) Binding of Cu(II) to the E2 domain of APP was recorded using electron paramagnetic resonance spectroscopy (EPR). Simulation of the EPR spectrum of Cu(II) bound to the E2 domain was performed using 4 nitrogen atoms as coordinating ligands for the 0.9 Cu(II)/E2 sample. The simulation parameters were: g_⊥_ = 2.053 and g_∥_ = 2.2528; line widths (gauss) were 11, 11, and 15; copper hyperfine splittings (gauss) were: 24, 24, and 176 gauss; nitrogen hyperfine splittings (gauss) were: 18, 18, and 11. The inserts show enlargements of part of the spectra which are marked by red lines. (B) The EPR intensity at 2650 gauss is plotted as a function of the amount of Cu(II) added to E2 domain. A 159 mM solution of E2 domain in 100 mM Mops 150 mM NaCl pH 7.0 was used.

To check the results of ITC measurements, we monitored binding of Cu(II) to the E2 domain using electron paramagnetic resonance (EPR) spectroscopy. Upon addition of Cu(II) to the E2 domain of APP an EPR signal with four lines centered at g value of 2.2528 appeared (Figure 3A). These peaks arise because of hyperfine coupling to the I = 3/2 Cu(II) nucleus. The EPR spectrum of the Cu(II) binding site of the E2 domain could be simulated assuming superhyperfine splitting (just barely resolved) in the perpendicular direction from four nitrogen ligands (Figure 3A). Thus, this binding event is associated to a specific Cu(II) binding site that is observed in the E2 domain using X-ray crystallography with four histidines as coordinating residues (His313, His382, His432, and His436 based on APP_695_ numbering) [30] (Figure 1C). As the amount of Cu(II) increased from 1.2 Cu(II) per E2 domain to 2.4 Cu(II) per E2 domain, the hyperfine pattern of the Cu(II) became more complex (Figure 3A) suggesting the presence of other Cu(II) binding sites with overlapping hyperfine structure except for the low-field peak around 2650 gauss. A plot of EPR intensity at this field strength versus the amount of Cu(II) added to E2 domain showed a stoichiometry of 0.9±0.1 for the first binding site (Figure 3B). Thus, the results of EPR spectroscopy confirmed that Cu(II) binds to the E2 domain at a specific site whose coordination sphere consists of four histidines.

### The E2 Domain does not Show Ferroxidase Activity in the Transferrin Assay

**Figure 4:**
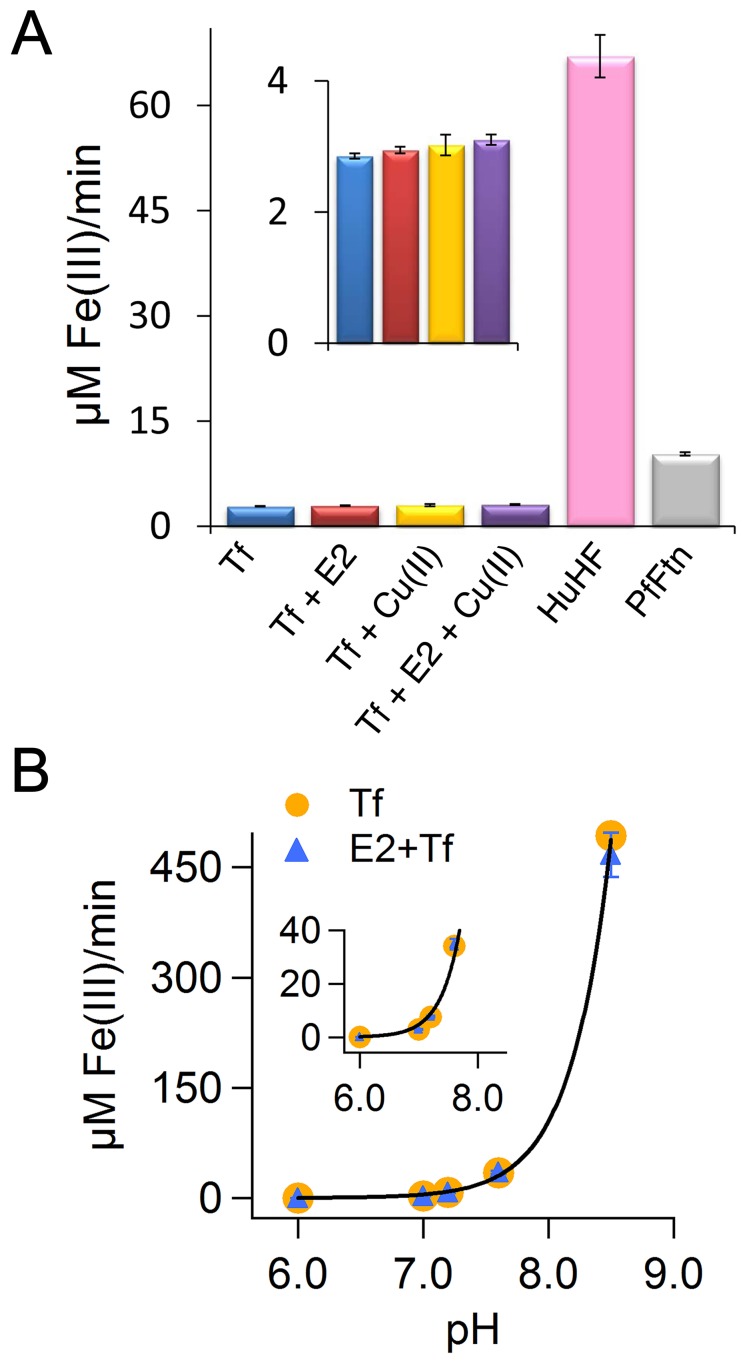
The E2 domain of APP does not have ferroxidase activity in transferrin assay. (A) The initial rate (µM Fe(III) formed per min) of Fe(III) formation was measured in the presence and absence of the E2 domain of APP using the transferrin assay. The effect of Cu(II) was tested on the ferroxidase activity of the E2 domain. The results were compared with the ferroxidase activity of HuHF and PfFtn. The initial rate of ferroxidase activity of ferritin was obtained from the initial slope of the progress curves at 310 nm for HuHF or at 315 nm for PfFtn. Concentrations of the E2 domain, HuHF (monomer) or PfFtn (monomer) were 1.6 µM. Measurements were performed at 37°C in 100 mM Mops, 100 mM NaCl pH 7.0. The concentrations of Fe(II) and of transferrin were 80 µM and 100 µM respectively. (B) The effect of pH on the ferroxidase activity of the E2 domain was measured and was compared with that of background oxidation of Fe(II) and incorporation of the Fe(III) product into transferrin. The concentrations of E2 domain, of transferrin, and of Fe(II) were 1.6 µM, 100 µM, and 80 µM, respectively. Measurements were performed at 37°C.

We showed that the results of Cu(II) binding to the E2 domain of APP were reproducible, which suggested to us that our E2 preparation was in a properly folded state. Subsequently, we checked if we can reproduce the ferroxidase activity of the E2 domain reported by Duce et al [21]. We measured the kinetics of Fe(II) oxidation by recording incorporation of the Fe(III) product into apo-transferrin and formation of the Fe(III)-transferrin complex at 460 nm (Figure 4A). We compared this activity with the ferroxidase activity of two ferritins, i.e. eukaryotic human H ferritin (HuHF) and archaeal *Pyrococcus furiosus* ferritin (PfFtn) as measured by following the formation of an Fe(III)-mineral core inside the cavity of these proteins in the absence of transferrin. Fe(III)-mineral core formation in HuHF was followed at 310 nm using a molar extinction coefficient of 2.47 mM^−1^cm^−1^ [31], [32], and for PfFtn at 315 nm using a molar extinction coefficient of 2.5 mM^−1^cm^−1^ [33] (Figure 4A). The transferrin assay was not used to measure the ferroxidase activity of ferritin because ferritin binds and stores the Fe(III) and therefore, the rate of transferrin-Fe(III) complex formation does not represents the actual rate of the ferroxidase activity of ferritin [29]. Both in the absence of the E2 domain and in its presence the rates of Fe(III)-transferrin complex formation were within experimental error identical; thus the E2 domain of APP (in the absence of the E1 domain) does not show ferroxidase activity in the transferrin assay (Figure 4A). These rates were within experimental error identical to those we observed previously in the presence or absence of the FD1 peptide [29]. In contrast, the ferroxidase activities of HuHF and PfFtn were significantly higher than the background oxidation of Fe(II) that was measured by the transferrin assay. The lower activity of PfFtn (Figure 4A) in comparison to that of HuHF at 37°C is because PfFtn is a hyperthermophilic protein which has its optimal activity at temperatures around 100°C. The E2 domain can bind to Cu(II) and this binding induces a large conformational change [30]. Therefore, we measured if binding of Cu(II) to the E2 domain can induce ferroxidase activity. We incubated the E2 domain with one Cu(II) per E2 domain and we looked for ferroxidase activity by measuring incorporation of the Fe(III) product into transferrin (Figure 4A). The results show that binding of Cu(II) does not induce ferroxidase activity in the E2 domain of APP. The presence of Cu(II) slightly increased the background oxidation of Fe(II) by molecular oxygen and incorporation of the Fe(III) product into transferrin in the presence or absence of the E2 domain (Figure 4A). Finally, we tested the effect of pH on the ferroxidase activity of the E2 domain (Figure 4B). The initial rate of background oxidation of Fe(II) and incorporation of the resultant Fe(III) product into transferrin increases hyperbolically as the pH increases from 6 to 8.5 consistent with our previous results [29]. Moreover, at none of the tested pH values the presence of the E2 domain increased the rate of Fe(II) oxidation above the background reaction (Figure 4B).

### The E2 Domain does not Consume Molecular Oxygen to Catalyze Oxidation of Fe(II)

**Figure 5:**
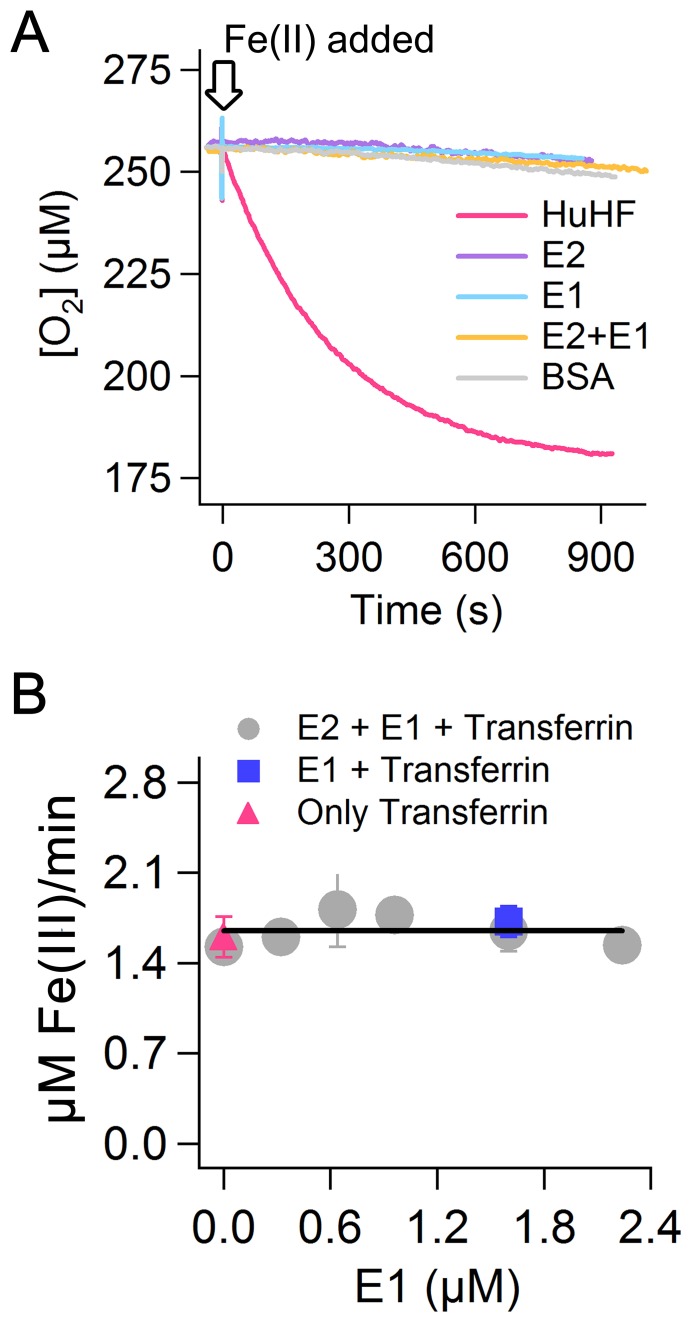
The E1 domain does not induce ferroxidase activity in the E2 domain of APP. (A) Consumption of molecular oxygen upon addition of Fe(II) was measured for HuHF (2.7 µM monomer), E2 domain (2.7 µM), E1 domain (2.7 µM), E2 domain (2.7 µM) in the presence of E1 domain (2.7 µM), and BSA (2.7 µM). The concentration of Fe(II) was 270 µM. Measurements were performed in 100 mM Mops, 100 mM NaCl pH 7.0. Temperature was 22°C. (B) The transferrin assay was used to measure the ferroxidase activity of the E2 domain (1.6 µM) in the presence of different amounts of E1 domain (gray circles), that of E1 domain alone (1.6 µM, blue rectangle), or that of background oxidation of Fe(II) and incorporation of the Fe(III)-product into transferrin in the absence of the E1 and E2 domain (purple triangle). In all experiments concentration of transferrin was 100 µM and that of Fe(II) was 80 µM. Temperature was 37°C.

To further test the proposed ferroxidase activity of the E2 domain of APP we recorded consumption of molecular oxygen which is the second substrate in the ferroxidase reaction. We compared the results of the E2 domain with those of HuHF and of BSA. HuHF consumes molecular oxygen to catalyze oxidation of Fe(II) and thus is used as a positive control. BSA is not able to catalyze oxidation of Fe(II) and is used as a negative control. HuHF shows significant consumption of dioxygen upon addition of 50 Fe(II) per subunit of protein (1200 Fe(II) per 24-mer) (Figure 5A). We found a stoichiometry of circa 3.5 Fe(II) per molecular oxygen consistent with the literature for Fe(II) added to HuHF in a ratio greater than 150 Fe(II) per 24-meric ferritin [34]. The activities of the E2 domain of APP and of BSA were zero the same as the FD1 peptide which we have tested previously [29]. Thus, consistent with the results obtained from UV-visible spectroscopy, we conclude that the E2 domain in the absence of the E1 domain does not catalyze oxidation of Fe(II) as measured on molecular-oxygen consumption.

### The E1 Domain does not Induce Ferroxidase Activity in the E2 Domain

Duce et al. [21] reported that the E1 domain stimulates the ferroxidase activity of the E2 domain circa two-fold to a level that is identical to that of recombinant soluble APP695α. Therefore, we measured the ferroxidase activity of the E2 domain of APP in the presence of different amounts of E1 domain using both the transferrin assay and dioxygen-consumption measurements. In the transferrin assay (Figure 5B), the presence of different amounts of the E1 domain did not affect the activity. Within experimental error the activity of the E2 domain was always identical to that of background oxidation of Fe(II) and incorporation of Fe(III) product into transferrin. Furthermore, the E1 domain alone also did not show any ferroxidase activity. Consistent with these data, oxygen consumption measurements also showed that in the presence of one E1 domain per E2, the activity is identical to that of the E2 domain alone and to that of BSA, i.e. zero (Figure 5A). Only HuHF as a positive control showed significant ferroxidase activity upon addition of Fe(II). Therefore, we conclude that the E1 domain does not activate the E2 domain for ferroxidase activity.

### The E2 Domain does not Bind Fe(II)

**Figure 6:**
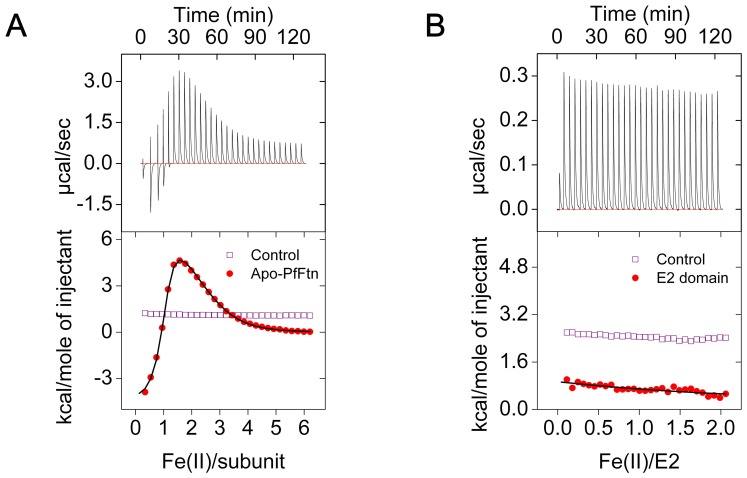
The E2 domain does not bind Fe(II). (A) Anaerobic binding of Fe(II) to *Pyrococcus furiosus* ferritin (PfFtn) was measured using isothermal titration calorimetry (ITC). Concentration of apo-PfFtn in the cell was 96 µM (subunit) and that of Fe(II) solution in the syringe was 9.18 mM. Measurements were performed at 25°C. A control experiment was performed in the absence of PfFtn to obtain the heat of dilution of Fe(II) titrated in buffer (purple rectangle). The data of integrated heat of binding of Fe(II) to apo-PfFtn (red circles) were corrected for the heat of dilution. The black line shows the fit of an equation with three sequential binding sites. A model of three sequential binding sites was required to obtain a fit to the data of integrated heat of binding. (B) Anaerobic binding of Fe(II) to the E2 domain of APP was measured by ITC. The concentration of the E2 domain in the cell was 40 µM and that of Fe(II) in the syringe was 1.27 mM. Measurements were performed at 25°C. A control experiment was performed in the absence of the E2 domain to obtain the heat of dilution of Fe(II) in buffer (purple rectangle). The data of integrated heat of binding of Fe(II) to the E2 domain (red circles) were corrected for heat of dilution. For all measurements buffer was 200 mM Mops, 150 mM NaCl, pH 6.9. Each experiment was performed at least in duplicate.

Because we found that the E2 domain of APP does not catalyze oxidation of Fe(II) using molecular oxygen and that the E1 domain does not induce any ferroxidase activity in the E2 domain, we tested if the E2 domain binds Fe(II) at all. We measured binding of Fe(II) to the E2 domain under anaerobic conditions using isothermal titration calorimetry (ITC) and we compared the results with those of Fe(II) binding to PfFtn as a positive control. Consistent with our previous observation [17], for PfFtn we observed three binding events per subunit (Figure 6A): one high affinity binding event with a stoichiometry of one and association constant of (9.00±0.4)·10^5^ M^−1^, and two lower affinity binding events each with a stoichiometry of one and an association constants of (3.3±0.2)·10^4^ M^−1^ and (1.4±0.1)·10^4^ M^−1^, respectively. These binding events have been assigned to binding of Fe(II) to the ferroxidase center and a gateway site in its vicinity [17]. The thermodynamic parameters of these bindings were within experimental error identical to our previous results [29]. Fe(II) binding to HuHF under anaerobic conditions also shows three binding sites the same as PfFtn [17]. For the E2 domain of APP (Figure 6B) however, within the sensitivity of the ITC experiments we did not observe significant binding of Fe(II) at pH 7.0. We found that the solution after ITC experiments turned milky suggesting aggregation of the E2 domain, which is possibly due to metal ion induced intermolecular interactions between E2 domains. This was possibly the reason for the observation of a small amount of heat consumed during the anaerobic Fe(II) titration (Figure 6B). This is in line with our observation that Fe(II) did not bind to APP-E2 crystals in soaking experiments the same as those reported by Dahms et al. ([30] and S.O.Dahms personal communication). Thus, in contrast to the data reported by Duce et al. [21] we conclude that the E2 domain of APP does not bind Fe(II) and it does not have a ferroxidase site.

### Conclusions

Our incentives for testing the ferroxidase activity of the E2 domain of APP were two observations: (1) we previously found that the FD1 peptide with the putative ferroxidase site of the E2 domain of APP does not have ferroxidase activity [29]. This result is inconsistent with the results reported by Duce et al. for the same peptide [21]. (2) We observed several internal inconsistencies in the data reported by Duce et al., more specifically: (i) The kinetic data for APP and for the E2 domain are not consistent with Michaelis-Menten kinetics (Figure 1 and 2 in reference [21]), yet K_M_ and V_max_ values are reported that were obtained from a fit of the Michaelis-Menten equation to the data (Figure S2). In the figures (Figure 1 and 2 in reference [21]) the fit to Michaelis-Menten equation is not shown but instead a non-hyperbolic hand-drawn fit to the data is shown. (ii) Two different rates are reported for the ferroxidase activity of the E2 domain in the absence of E1 domain, namely 10 µM Fe(III) formed per minute (Figure 2A, ref. [21]) and 16 µM Fe(III) formed per minute (Figure 2E, ref. [21]). This suggests an uncertainty of greater than 50%. However, error bars in each figure show an error of less than 1%. (iii) The ferroxidase activity of the E2 domain is reported to be circa 3 times less than the ferroxidase activity of APP but the quoted k_cat_ for the E2 domain is within experimental error identical to that of the full-length protein. (iv) The concentration of buffer after addition of Fe(II) and protein was less than 10 mM. This concentration of buffer has a low buffering capacity and may result in significant pH changes. As we have shown here and previously [29], small changes in pH drastically affect the background oxidation of Fe(II) and incorporation of the resulting Fe(III) product into transferrin.

We tested the ferroxidase activity of the E2 domain of APP and the effect of the E1 domain on this activity. With two independent methods, i.e. transferrin-assay and dioxygen-consumption measurements, we did not observe any ferroxidase activity for the E2 domain of APP neither in the presence nor the absence of the E1 domain. We also tested if binding of Cu(II) to the E2 domain can induce a ferroxidase activity in analogy to the ferroxidase activity of multicopper oxidases such as ceruloplasmin. Cu(II) was shown to bind to the E2 domain but it did not induce any ferroxidase activity. We further investigated Fe(II) binding to the E2 domain by ITC and did not see any heat resulting from an interaction with Fe(II). Because a purely entropy-driven binding event is very unlikely, the E2 domain probably does not bind Fe(II).

Our observation that the E2 domain of APP does not possess ferroxidase activity raises the question if differences in the protein purification procedure that we use here and those that were used by Duce et al. have influenced the results. Firstly, in the procedure that was used by Duce et al. [21] after purification of the proteins, a metal ion chelator, i.e. N, N, N′, N′-tetrakis (2-pyridylmethyl) ethylenediamine (TPEN) was added to remove possible free Zn(II). TPEN is known to bind different metal ions including Fe(II) [35], [36]. This chelator has not been removed and it is possible that the presence of free TPEN has facilitated oxidation of Fe(II) the same as facilitation of the Fe(II) oxidation by metal ion chelator ethylenediaminetetraacetic acid (EDTA) [37]. In contrast to Duce et al. in our protein purification step we did not use any metal ion chelator. Secondly, Duce et al. overexpressed the E2 domain of APP in *E. coli* and the E1 domain was expressed in yeast Pichia pastoris. In contrast both the E1 and E2 domains were overexpressed in *E.coli* in our study. As in both cases the E2 domain derives from *E. coli* and as no glycosylation site has been reported for the E1 domain, the discrepancy between our results and the results reported by Duce et al. on the ferroxidase activity of the E2 domain cannot be because of the differences in the protein expression procedures.

In conclusion, in our experiments we did not observe any direct biochemical indications to support the view that ferroxidase activity is associated with the E1 or E2 domains of APP. Because the putative ferroxidase site of the E2 domain is present in all APP isoforms and the FD1 which lacks the ferroxidase activity [29], we conclude that the E2 domain of APP is not a ferroxidase and the cell-biology experiments performed by Duce et al. and the proposed functioning of APP as a ferroxidase in Alzheimer disease must be re-evaluated. APP may interact with ferroportin to stabilize this protein and facilitate Fe(II) export, or it may interact with other proteins which are involved in Fe(II) oxidation. Hephaestin has been recently shown to be present in neurons [38], [39]. It is a membrane protein and a ceruloplasmin homologue [40] that has ferroxidase activity [41]. Thus, if the ferroxidase activity of a protein is required in neurons, instead of APP, hephaestin may function as a ferroxidase and facilitates iron export functioning of ferroportin the same as ceruloplasmin [14]. It has been shown that hephaestin and ceruloplasmin are essential for iron homeostasis in central nervous system [42].

## Materials and Methods

### Chemicals

All chemicals were reagent grade and were purchase from Sigma Aldrich.

### Preparation of Proteins

Apo-PfFtn and apo-HuHF were prepared as described previously [17]. The protein concentration was measured with the BCA assay using bovine serum albumin as standard. The APP-E2 and APP-E1 domains were prepared essentially as described before [30], [43], [44]. In short: APP-E1 and E2 were expressed in *E. coli* and initially purified via Ni-affinity chromatography on a His-Trap FF crude column (GE-healthcare). The his-tag was cleaved off from E1 and E2 using V8 protease (Calbiochem/Merck) at pH 5.7 and 8.0, respectively. Both, the V8-protease and the cleaved his-tag, were removed afterwards. In the case of APP-E1, the cleaved his-tag and E1 were unspecifically bound to the Ni-column at low salt to remove V8 protease in the flow-through. E1 and cleaved his-tag were then eluted separately by high NaCl and imidazole, respectively. To separate APP-E2 from V8 and the cleaved his-tag, the latter was bound to a Ni-column, whereas E2 was captured on a heparin column and eluted by a salt gradient. Final polishing of both domains was performend by gel filtration on a Superdex 75 10/300 column (GE Healthcare) in a buffer containing 150 mM NaCl, 5 mM tris, pH 8.0. Both proteins eluted a homogenous peak. The fractions used for the subsequent experiments are shown in Figure S1 before pooling.

### Preparation of Apo-transferrin

Apo-bovine transferrin (>98% pure) was purchased from Sigma Aldrich. The lyophilized powder was dissolved in working buffer (100 mM Mops, 100 mM NaCl, pH 7.0). Subsequently, to remove possible metal complexing agents that may have remained in the powder from the manufacturer production procedure, we washed the protein in consecutive dilution and concentration steps. This was performed in 400 ml of working buffer using an ultrafiltration membrane with a cut-off of 10 kDa (Millipore). Finally, the protein concentration was measured with the BCA assay using bovine serum albumin as standard.

### Steady State Kinetics of Fe(III) Formation for PfFtn and HuHF

The initial rates (µM Fe(III) formed/min) were obtained from the initial slope of the progress curves of Fe(III) formation which were recorded at 310 nm for HuHF (ε_310nm_ = 2.47 mM^−1^cm^−1^ for Fe(III) in HuHF [32]) or at 315 nm for PfFtn (ε_315nm_ = 2.5 mM^−1^cm^−1^ for Fe(III) in PfFtn [33]) on a fiber-optics spectrophotometer (Avantes). The reaction was started by addition of 5 µl anaerobic solution of FeSO_4_ (16 mM) to the reaction cuvette (1 ml glass cuvette) containing aerobic buffer (992 µl), and HuHF or PfFtn (3 µl). Before addition of FeSO_4_ solution, the spectrophotometer was blanked using the mixture of protein in buffer. The final concentration of HuHF (monomer) or PfFtn (monomer) was 1.6 µM. Temperature was set to 37°C.

### Transferrin Assay

The ferroxidase reaction for E2 domaion of APP was measured by recording the progress curves of transferrin-Fe(III) complex formation. The progress curves were recorded on a fiber-optics spectrophotometer (Avantes). The reaction was started by addition of 5 µl anaerobic solution of FeSO_4_ (16 mM) to the reaction cuvette (1 ml glass cuvette) containing buffer (783-718 µl), E2 domain (50 µl) and/or E1 domain (0–65 µl), transferrin (167 µl). Before addition of FeSO_4_ solution, the spectrosphotometer was blanked using the mixture of protein in buffer. Final concentration of E2 domain was 1.6 µM and that of transferrin was 100 µM. For experiments at pH 7.0 or 7.2 the buffer was 200 mM Mops, 100 mM NaCl, for experiments at pH 6.0 the buffer was 200 mM Mes, 100 mM NaCl, for experiments at pH 7.6 the buffer was 200 mM Hepes, 100 mM NaCl, and for experiments at pH 8.5 the buffer was 200 mM Tris, 100 mM NaCl. Initial rate of Fe(III) formation was calculated from the initial slope of the progress curves using a molar extinction coefficient for diferric transferrin complex of ε_460 nm_ = 4.56 mM^−1^cm^−1^ [45]. Measurements were performed at 37°C.

### Oxygen Consumption Measurements

The consumption of molecular oxygen was measured amperometrically using a Clark electrode [46]. The ferroxidase reaction for E2 domain was started by addition of 5 µl of anaerobic solution of FeSO_4_ (108 mM) to the Clark electrode cell (2 ml volume) containing buffer (1795–1895 µl), E2 domain (100 µl) and/or E1 domain (100 µl). The ferroxidase reaction for the HuHF or BSA was started by addition of 5 µl anaerobic solution of FeSO_4_ (108 mM) to the Clark electrode cell (2 ml volume ) containing buffer (1983 µl), BSA (12 µl) or HuHF (12 µl). Buffer was 200 mM Mops, 100 mM NaCl, pH 7.0. Temperature was 25°C.

### Metal Binding to Ferritin and E2 Domain of APP

Cu(II) binding experiments under aerobic conditions and anaerobic Fe(II) binding experiments were performed using isothermal titration calorimetry with a VP-ITC microcalorimeter (GE-healthcare) as described before [17]. For Cu(II) binding to the E2 domain we first prepared a 1.27 mM CuSO_4_ solution in 100 mM Mops, 150 mM NaCl pH 5.3. The pH of CuSO_4_ solution was kept at 5.3 because some precipitation was observed at pH 7.0. The E2 domain was washed with 200 mM Mops, 150 mM NaCl, pH 7.0 using an ultrafiltration membrane with a cut-off of 10 kDa (Millipore). A control experiment in the absence of E2 domain using the working buffer was performed to obtain the heat of dilution. To perform anaerobic titration of Fe(II) to apo-PfFtn or to the APP-E2 domain, the ITC machine was placed in a polyethylene bag (Atmosbag, Sigma Aldrich) [17]. Apo-PfFtn and the E2 domain were washed with 200 mM Mops, 150 mM NaCl, pH 7.0 using an ultrafiltration membrane with a cut-off of 10 kDa (Millipore). Subsequently, solutions were made anaerobic with more than 20 cycles of purging high purity argon gas (99.999% pure) and vacuuming. The anaerobic solutions were put in the polyethylene bag and were kept sealed using a rubber septum. Anaerobic solutions of FeSO_4_ (9.18 mM or 1.27 mM) at pH of 3.6 were also prepared and placed in the polyethylene (PE) bag. The FeSO_4_ solution was kept closed during the experiments. An anaerobic atmosphere in the PE bag was established by at least five argon/vacuum cycles and finally the PE bag was filled with argon. During all experiments an overpressure of argon was kept. Anaerobic solutions were taken out from the sealed vessels using a gas tight syringe. Measurements were performed at 25°C by titrating 9.18 mM or 1.27 mM Fe(II) into 96 µM PtFtn or 40 µM APP-E2, respectively. Control experiments, i.e. in the absence of apo-PfFtn or E2 domain, were performed to obtain the heat of dilution. ITC data measured by 20 injections (Cu(II) titration) or 30 injections (Fe(II) titration) of 4.5 µl each (Cu(II) titration) or 3 µL each (Fe(II) titration) (first injection was only 2 µL and the respective data point was excluded from the processing) with 240 seconds delay between the injections. The cell content was stirred at 307 rpm. Analysis of the ITC data was performed using Origin 7.0 software.

### Electron Paramagnetic Resonance Spectroscopy (EPR) Measurements

Spectra were recorded with a Bruker ECS-106 EPR spectrometer with the ‘Swedish’ dewar system for sample cooling [47]. The spectrometer settings were: microwave power, 2.01 mW; modulation amplitude, 5.069 gauss; modulation frequency 100 kHz; microwave frequency 9,187 MHz; Temperature, 102 K. The simulation was done as described previously [48].

## Supporting Information

Figure S1 **15% SDS-PAGE showing the collected fractions of the final gel filtration step of the respective E2- (A) and E1- (B) purification used for the herein described experiments.** The molecular weight of the marker proteins (left column of each gel) is given in kDa on the left of the two panels.
(TIF)

Figure S2 **The simulation shows our attempt to fit a Michaelis-Menten equation into the data reported by Duce et al. (Cell (2010)142∶857–867) for the ferroxidase activity of APP695α.** The fit to the data shows the best possible fit that was obtained using Igor-pro software. The simulation shows that the data cannot be fitted with Michaelis-Menten equation.
(TIF)
