# Supplementary figures and images for: The Amyloid Precursor Protein (APP) Does Not Have a Ferroxidase Site in Its E2 Domain
Source: PLoS One. 2013 Aug 19;8(8):e72177. doi: 10.1371/journal.pone.0072177 (PMC3747053; doi:10.1371/journal.pone.0072177)

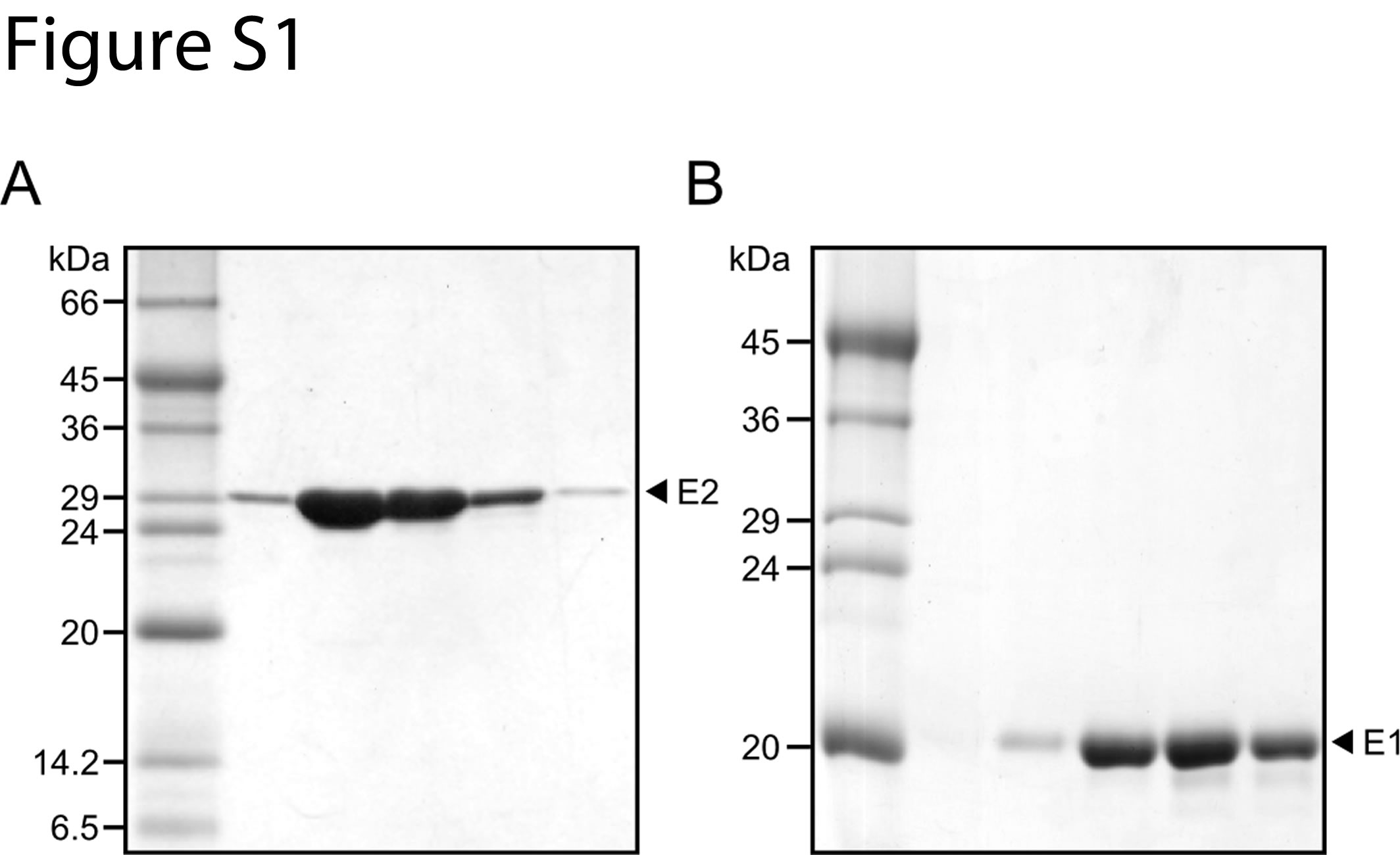

Supplement: Figure S1 — 15% SDS-PAGE showing the collected fractions of the final gel filtration step of the respective E2- (A) and E1- (B) purification used for the herein described experiments. The molecular weight of the marker proteins (left column of each gel) is given in kDa on the left of the two panels. (TIF) [file pone.0072177.s001.tif]

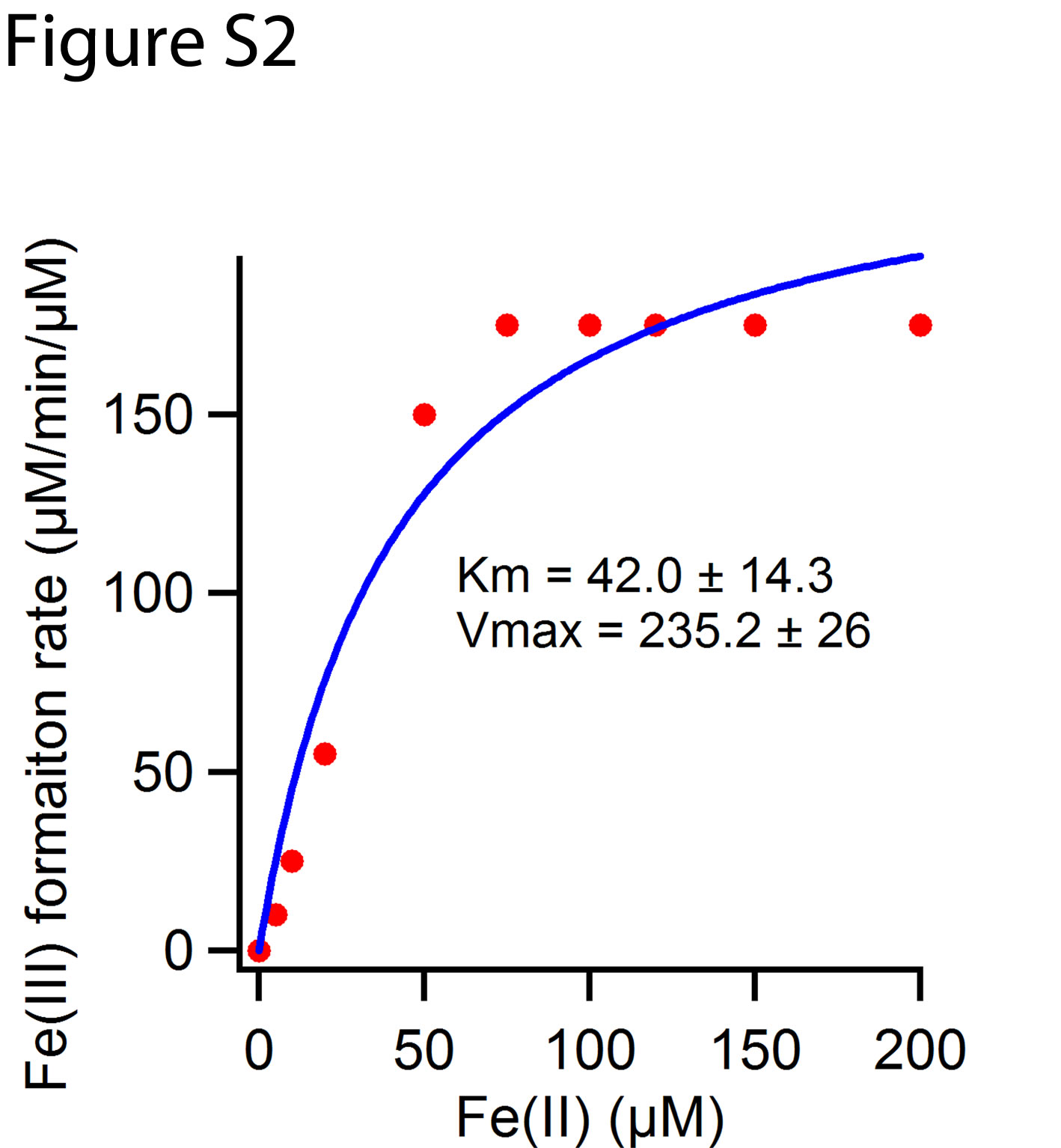

Supplement: Figure S2 — The simulation shows our attempt to fit a Michaelis-Menten equation into the data reported by Duce et al. (Cell (2010)142∶857–867) for the ferroxidase activity of APP695α. The fit to the data shows the best possible fit that was obtained using Igor-pro software. The simulation shows that the data cannot be fitted with Michaelis-Menten equation. (TIF) [file pone.0072177.s002.tif]
